# Editorial: Development and function of GABAergic interneurons in physiology and pathologies

**DOI:** 10.3389/fnmol.2024.1412738

**Published:** 2024-05-03

**Authors:** Milena Cannella, Maria Vincenza Catania, Ferdinando Nicoletti

**Affiliations:** ^1^Mediterranean Neurological Institute Neuromed (IRCCS), Pozzilli, Italy; ^2^Institute for Biomedical Research and Innovation (IRIB), National Research Council (CNR), Catania, Italy; ^3^Sapienza University of Rome, Rome, Lazio, Italy

**Keywords:** neurodevelopment, GABAergic interneurons, CNS, cortex, hippocampus

The purpose of this Research Topic was to collect articles that capture a snapshot of the state of the art in this leading topic in neuroscience, i.e., the role of cortical and hippocampal GABAergic interneurons in network oscillations and behavior in physiology and pathology, with special focus on stress-related disorders and neuroinflammatory disorders. The Research Topic includes two mini-reviews, one review, and three research articles.

The first mini-review (Aksenov et al.) describes the fundamental role of interneurons in shaping the developmental trajectories of the CNS, in relation to oxygen supply. Interestingly, interneuron subtypes expressing vasoactive intestinal peptide (VIP) or somatostatin (SST) regulate brain microvessels during adolescence, whereas interneurons expressing nitric oxide synthase (NOS) regulate microvessels earlier in development. Regulation of microvessels by these interneurons is not homogenous, with activation of VIP-expressing interneurons causing vasodilation, activation of SST-expressing interneurons causing vasoconstriction, and activation of NOS-expressing interneurons displaying both functions in a context-dependent manner. Knowing that the activity of interneurons may be critically affected by stressful events, this novel form of regulation of the neurovascular unit may raise new testable hypotheses of how stress causes changes in network activity and brain function. Thus, interneurons become potential targets for novel strategies aimed at optimizing oxygen and nutrient supply in CNS disorders.

The second mini-review is from a leading group in preclinical and clinical studies on stress-related disorders (Bigio et al.). The Authors describe the role of epigenetics in the long-lasting outcome of early life stress on brain function. They focus on the role of the emotional portion of the hippocampus (ventral hippocampus in rodents and anterior hippocampus in humans) in stress-related disorders and offer an elegant description of how epigenetic mechanisms affect different forms of neuroplasticity. This has a high translational value because it explores the mechanisms by which adverse childhood experiences produce a lifelong vulnerability to psychiatric disorders. As the Authors state, “while it is not possible to roll back the clock” a better understanding of the mechanisms linking early life stress to the long-lasting neurochemical and behavioral outcome “may provide a path for compensatory neuroplasticity toward more positive health directions.” Mitochondria may represent a valuable target for novel treatments in psychiatric disorders including stress-related disorders.

The review of Mueller-Buehl et al. focuses on the role of perineuronal nets (PNNs) enwrapping parvalbumin-positive (PV^+^) interneurons in the regulation of E/I balance and network activity in the cerebral cortex. PNNs are specialized structures of the extracellular matrix formed by complex proteoglycans (e.g., aggrecan, neurocan, brevican) linked to chondroitin sulfate, PNNs are formed during early postnatal development in a period that coincides with the closure of the temporal windows of cortical plasticity. PNNs retain their plasticity also in adult life, as suggested by the increase in PNN density in the somatosensory cortex associated with chronic pain (Mascio et al., 2022). The Authors offer an elegant description of how changes in PNN density are associated with a variety of CNS disorders, including schizophrenia, Alzheimer's disease, multiple sclerosis, and epilepsy. This is a novel exciting field in neuroscience, which, in the near future, may help to find a way to manipulate the E/I balance by inducing changes in the formation and/or degradation of PNNs.

Many disorders are characterized by marked changes in neuroplasticity, including the E/I balance and activity-dependent synaptic plasticity. These also include neuroinflammatory disorders, such as multiple sclerosis (MS). Adinolfi et al. provide evidence that experimental autoimmune encephalomyelitis (EAE) in mice (an established experimental animal model of MS) is associated with alterations in the hippocampal GABAergic circuit. Interestingly, EAE caused downregulation of *Sam68* selectively in the CA3 and dentate gyrus (DG) hippocampal subregions. Sam68 is a ubiquitous multifunctional RNA binding protein involved in different biological mechanisms such as mRNA transport, translation, and splicing. As a result, the inclusion of exon 11a of the *Arhgef9* gene encoding a postsynaptic protein playing an essential role in GABAergic synapses was selectively increased in the CA3 region of EAE mice. What makes these findings particularly exciting is the evidence that mutations of the protein encoded by the *Arhgef9* gene are associated with cognitive impairment and epilepsy. These findings are exciting because they strengthen the link between GABAergic interneurons and synaptic dysfunction and cognitive impairment in MS and other neuroinflammatory disorders.

The elegant manuscript by Li et al. suggests a role for PV^+^ interneurons in the infralimbic cortex (a portion of the medial prefrontal cortex in mice) in aggression induced by social isolation early in life. Interestingly, chemogenic inactivation of PV^+^ interneurons reproduced the effect of early social isolation in house-grouped mice (i.e., in control mice). In addition, social isolation caused changes in the number and activity of PV^+^ interneurons (in addition to changes in dendritic structures and spine morphology of pyramidal neurons) in the medial prefrontal cortex. Aggression during adolescence is an emergent social problem, particularly after COVID-19 pandemic. Understanding the neural mechanisms that regulate aggression in response to social isolation may provide new potential therapeutic targets.

The elegant research article by Zhang et al. focuses on the role of the transcriptional coactivator of the peroxisome proliferator-activated receptor 1α (PGC-1 α) in the regulation of cortical plasticity. PGC-1 α is a key regulator of mitochondrial biogenesis, one of the leading mechanisms underlying mitochondrial quality control. Using PGC-1 α knockout mice the Authors have demonstrated that PGC-1 α plays a key role in the opening and closure of the critical window of cortical plasticity and that the lack of PGC-1 α during development induces a long-lasting dysregulation of PV^+^ interneurons and associated PNN structures, thus recapitulating a schizophrenia-like phenotype. What makes the article extremely interesting is that an inhibitor of matrix metalloproteinases (the enzymes degrading PNNs) corrected abnormalities in developmental cortical plasticity and synaptic ultrastructures in PGC-1 α knockout mice. This raises the attractive possibility that PNNs can be targeted to correct abnormalities in cortical plasticity underlying schizophrenia and other psychiatric disorders.

It has been a privilege to edit these excellent research articles and reviews.

## Author contributions

MC: Writing – review & editing. MVC: Writing – review & editing. FN: Writing – review & editing.
